# Racial differences in predictive value of the 21-gene recurrence score assay: a population-based study using the SEER database

**DOI:** 10.1007/s12282-022-01371-z

**Published:** 2022-05-26

**Authors:** Jiwoong Jung, Ki-Tae Hwang, In Sil Choi, Byoung Hyuck Kim, Sohee Oh, Jongjin Kim, Jeong Hwan Park, Jin Hyun Park, Se Hyun Paek, Sook Young Jeon, Tae-Hoon Yeo

**Affiliations:** 1 Department of Surgery, Uijeongbu Eulji Medical Center, Eulji University School of Medicine, Uijeongbu, Republic of Korea; 2 Department of Surgery, Seoul National University College of Medicine, 39, Boramae-Gil, Dongjak-gu, Seoul, 156-707 Republic of Korea; 3 Department of Surgery, Seoul Metropolitan Government Seoul National University Boramae Medical Center, 39, Boramae-Gil, Dongjak-gu, Seoul, 156-707 Republic of Korea; 4 Department of Internal Medicine, Seoul Metropolitan Government Seoul National University Boramae Medical Center, Seoul, Republic of Korea; 5 Department of Radiation Oncology, Seoul Metropolitan Government Seoul National University Boramae Medical Center, Seoul, Republic of Korea; 6 Medical Research Collaborating Center, Seoul Metropolitan Government Seoul National University Boramae Medical Center, Seoul, Republic of Korea; 7 Department of Pathology, Seoul Metropolitan Government Seoul National University Boramae Medical Center, Seoul, Republic of Korea; 8 Department of Surgery, College of Medicine, Ewha Womans University, Seoul, Republic of Korea; 9 Department of Surgery, Hallym University Kangnam Sacred Heart Hospital, Seoul, Korea; 10 Department of Surgery, Seoul Medical Center, Seoul, Republic of Korea

**Keywords:** Breast cancer, Chemotherapy, 21-gene recurrence score assay, Race

## Abstract

**Purpose:**

The 21-gene recurrence score (RS) assay is currently used for predicting chemotherapeutic benefits for hormone receptor-positive (HR +) early-stage breast cancer patients without consideration regarding racial differences in that predictive value. This study aimed at demonstrating racial differences in the predictive values of the 21-gene RS assay.

**Methods:**

The study cohort was selected from the Surveillance, Epidemiology, and End Results (SEER) database. Breast cancer-specific mortality (BCSM) was compared between patients who received chemotherapy (the “CTx group”) and those who did not (the “no CTx group”) to estimate the predictive value of the assay. This comparison was repeated for each racial group.

**Results:**

Among 88,498 T1 − 2N0 HR + breast cancer patients who had results of 21-gene RS, 13,123 patients had RS > 25, which included 10,697 Whites, 1282 Blacks, and 1,144 Asian Americans/Pacific Islanders (AAPIs). Chemotherapy was administered to 8364 patients (63.4%). The adjusted hazard ratio for BCSM in the CTx group (vs. no CTx group) was 0.734 (95% confidence interval [CI] 0.588–0.917) in Whites, 0.748 (95% CI 0.428–1.307) in Blacks, and 1.343 (95% CI 0.558–3.233) in AAPIs. No subgroup within patients with RS > 25 among non-White women showed a significant predictive value of the 21-gene RS assay, except for Black women with grade 3 tumors.

**Conclusion:**

The predictive value of the 21-gene RS assay for assessing chemotherapy benefit was validated in White women based on the SEER database, although the predictive value was not warranted in non-White women.

**Supplementary Information:**

The online version contains supplementary material available at 10.1007/s12282-022-01371-z.

## Introduction

Multigene expression assays have become an integral component of treatment planning for women with hormone receptor (HR)-positive and node-negative early breast cancer (EBC). The 21-gene recurrence score (RS) assay (Oncotype DX, Genomic Health, Redwood City, USA) is the only multigene assay validated as both a prognostic and predictive tool of chemotherapy benefit in these patients.

This assay was developed and validated based on the clinical and genetic background of tumors from a subset of participants enrolled in the B-20 and B-14 National Surgical Adjuvant Breast and Bowel Project (NSABP) trials [1]. In principle, a well-developed biomarker should be discovered and validated in a cohort that is representative of the populations targeted for its clinical application [2]. The subset of participants from the two NSABP trials used to develop the RS was assumed to include only 5–6% of Black women [1, 3, 4]. Given that the participants of those trials were enrolled in the 1980s and the early 1990s from institutions in the United States and Canada [3, 4], Asian participants were likely to be as small in number as Black women.

The under-representation of women from racial minority groups in the establishment of the RS raises questions about the clinical relevance of the 21-gene RS assay in populations other than White people. Given the known racial disparities in the characteristics of breast cancer between White and non-White women [5–8], it is important to examine the performance of the 21-gene RS assay in diverse patient populations to validate the current approach for tailoring treatment for women from racial minority groups.

This study aimed at examining the performance of the 21-gene RS assay in predicting the benefits of chemotherapy among non-White women.

## Methods

### Study design and subjects

This study was designed as a population-based, retrospective cohort study using the Surveillance, Epidemiology, and End Results (SEER) Oncotype DX Database [9]. This specialized database includes results from the 21-gene RS assay that are provided by linkage of test orders and results from the Genomic Health Clinical Laboratory with invasive breast cancer cases in the SEER registry diagnosed between 2004 and 2015, with follow-up for survival through December 31, 2016. The study followed the Strengthening the Reporting of Observational Studies in Epidemiology (STROBE) reporting guidelines.

We identified women with HR-positive, node-negative T1–T2 invasive breast cancers from the SEER Oncotype DX Database using SEER*Stat 8.3.6 software (Supplementary Figure S1). Information on age at diagnosis, race, year of diagnosis, AJCC stage, T category, histologic grade, histologic type, HR status, human epidermal growth factor receptor 2 (HER2) status, surgery, and receipt of radiotherapy (RT) and any chemotherapy as part of the first course of therapy was collected from SEER records. Patients who had the RS variable as a continuous measure (0–100 points scale) were grouped in the RS categories established for the Trial Assigning Individualized Options for Treatment (TAILORx) because current guidelines for breast cancer are based on those cutoffs [10, 11]. Patients were divided into low-risk (RS: 0 − 10), intermediate-risk (RS: 11 − 25), and high-risk (RS > 25) groups. Women with no race information and longitudinal follow-up for survival status information were excluded from the study. The primary outcome was defined as breast cancer-specific mortality (BCSM), based on a “breast”-related cause in the SEER dataset. Deaths from other causes were assumed to be censored at the time of death.

### Sensitivity analysis

The 21-gene RS-based risk grouping initially defined the high-risk group as patients who had tumors with RS > 30 [1, 12], although it was expanded to include those who had tumors with RS > 25 after the TAILORx report. Therefore, we performed a sensitivity analysis with the initial criteria of the high-risk group (RS > 30) to address a potential discrepancy between these two high-risk criteria. The corresponding risk estimates were calculated in the same way as in the main analysis.

### Propensity score matching

The effects of selection bias were minimized by matching propensity scores (PSs), which were calculated using a logistic regression model with the selection of chemotherapy as the dependent variable and other variables that were selected based on their univariate associations with the use of chemotherapy. Logistic regression model for PS calculation included the following independent variables: age (≤ 50 vs. > 50 years), T category, histologic grade, hormonal status, HER2 status, type of breast surgery and receipt of radiotherapy. Patients from the two groups divided by chemotherapeutic use were paired 1:1 using nearest-neighbor matching with a caliper width less than 0.25 standard deviations. Standardized differences were estimated before and after the matching to evaluate the covariates’ balance, with absolute values of < 0.1 considered indicative of well-balanced groups [13]. These analyses were performed with R software version 3.5.2.

### Statistical analyses

The characteristics of the two groups were compared using the chi-square test and two-sample *t* test. Survival curves were compared using the Kaplan–Meier method and log-rank test. Cox’s proportional hazard regression models were used to calculate hazard ratios (HRs) and 95% confidence intervals (CIs) for the associations of BCSM with the prognostic variables and treatments. All tests were two-sided, and *P* values ≤ 0.05, were considered statistically significant. The analyses were performed using IBM SPSS software (version 20.0; IBM Corp., Armonk, NY, USA).

## Results

### Characteristics of the study cohort

**Fig. 1:**
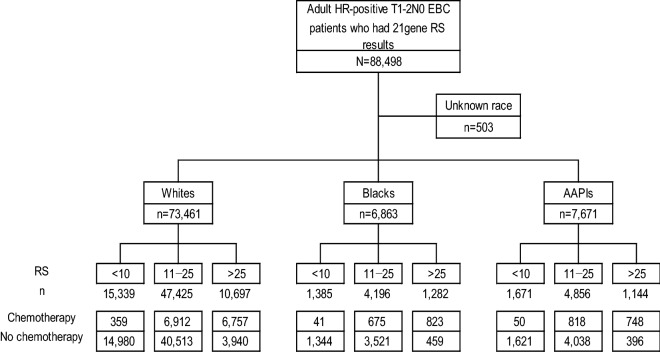
Overview of the 21-gene RS-based risk categorization and treatment of chemotherapy within each racial group. *AAPIs* Asian American/Pacific Islanders, *EBC* early breast cancer, *HR* hormone receptor, *RS* recurrence score

We identified 88,498 women who had HR-positive T1–T2N0 breast cancer and the 21-gene RS result for this study. Overall, 503 women with no racial information were excluded. The majority of the remaining 87,995 were White women (*n* = 73,461). Only 6863 and 7671 women were Blacks and Asian American/Pacific Islanders (AAPIs) (Fig. 1). More than 80% of the entire cohort had an RS of ≤ 25. Among the Whites, 20.9% had RS ≤ 10, 64.6% had RS between 11 and 25, and 14.6% had RS > 25. The distribution of 21-gene RS-based risk categories in AAPIs was similar to that in Whites (21.8%, 63.3%, and 14.9%, respectively). It is noteworthy that women who had RS > 25 were more represented in the Black population (18.7% vs. 14.6% in Whites and 14.9% in AAPIs).

The number of patients who had RS > 25, who would benefit from adjuvant chemotherapy based on the TAILORx results, was 13,123, which included 10,697 Whites, 1282 Blacks, and 1144 AAPIs (Table 1). Most of the 1,144 AAPIs were Asians, and the detailed ethnicities are listed in supplementary Table S1. Blacks and AAPIs were younger at diagnosis and had higher grade and larger tumors than Whites in this sub-cohort. The proportion of progesterone receptor-positive tumors was slightly lower in Blacks and AAPIs (71.5% in Blacks and 71.9% in AAPIs vs. 73.1% in Whites), although more than 99% of patients had estrogen receptor-positive tumors, regardless of race among the entire sub-cohort. Chemotherapy was administered to 63.5% of this sub-cohort, although the actual rate would be higher than our results because the SEER dataset only includes incomplete information regarding adjuvant therapies. We did not observe statistically significant differences in the proportion of patients receiving chemotherapy between the racial groups. RT treatments were less frequently administered in AAPIs than in Whites and Blacks (48.8% vs. 53.5% in Whites and 53.4% in Blacks), which might reflect more frequent mastectomies among AAPIs (40.7% vs. 33.1% in Whites and 31.7% in Blacks). The median follow-up period was 48 months for the entire sub-cohort, and was shorter in Blacks and AAPIs than in Whites (43 and 46 months, respectively, vs. 49 months in Whites). During this period, 409 (3.1%) deaths were related to breast cancer in Whites and 25 (2.2%) in AAPIs. The number of breast cancer-related deaths was 52 (4.1%) in Blacks, which was more frequent than in Whites and AAPIs despite a shorter follow-up period.

**Table 1:** Baseline characteristics of each racial group within the 21-gene RS-based high-risk group (RS > 25) selected from the SEER database

	Total	Whites	Blacks	AAPIs	*P*-value
	No	No. (%)	No. (%)	No	
	13,123	10,697	1,282	1,144	
Year of diagnosis					< 0.001
2004–2006	916	802	56	58	
2007–2009	3,360	2,783	306	271	
2010–2012	4,275	3,455	416	404	
2013–2015	4,572	3,657	504	411	
Patient age, years (range)	59 (18–92)	60 (18–91)	56 (24–92)	56 (21–87)	
≤ 50	3,165	2,437 (22.8%)	388 (30.3%)	340 (29.7%)	< 0.001
> 50	9,958	8,260 (77.2%)	894 (69.7%)	804 (70.3%)	
T category					0.001
T1	9,270	7,628 (71.3%)	882 (68.8%)	760 (66.4%)	
T2	3,853	3,069 (28.7%)	400 (31.2%)	384 (33.6%)	
Surgery type					< 0.001
BCS	8,705	7,151 (66.9%)	876 (68.3%)	678 (59.3%)	
Mastectomy	4,418	3,546 (33.1%)	406 (31.7%)	466 (40.7%)	
Histologic type					0.004
IDC	10,898	8,847 (82.7%)	1,092 (85.2%)	959 (83.8%)	
ILC	769	654 (6.1%)	55 (4.3%)	60 (5.2%)	
IDC + ILC	675	577 (5.4%)	47 (3.7%)	51 (4.5%)	
Others	781	619 (5.8%)	88 (6.9%)	74 (6.5%)	
Histologic grade					< 0.001
1	1,118	959 (9.0%)	82 (6.4%)	77 (6.7%)	
2	5,725	4,709 (44.0%)	524 (40.9%)	492 (43.0%)	
3	6,067	4,848 (45.3%)	654 (51.0%)	565 (49.4%)	
Unknown	213	181 (1.7%)	22 (1.7%)	10 (0.9%)	
HR status					0.28
ER + PR +	9,487	7,762 (72.6%)	906 (70.7%)	819 (71.6%)	
ER + PR −	3,550	2,865 (26.8%)	366 (28.5%)	319 (27.9%)	
ER + unknown PR	22	20 (0.2%)	0 (0.0%)	2 (0.2%)	
ER − PR +	64	50 (0.5%)	10 (0.8%)	4 (0.3%)	
HER2 status					0.001
Negative	8,081	6,508 (60.8%)	825 (64.4%)	748 (65.4%)	
Positive	433	346 (3.2%)	51 (4.0%)	36 (3.1%)	
Unknown	4,609	3,843 (35.9%)	406 (31.7%)	360 (31.5%)	
Radiotherapy	6,961 (53.0%)	5,718 (53.5%)	685 (53.4%)	558 (48.8%)	0.01
Chemotherapy	8,328 (63.5%)	6,757 (63.2%)	823 (64.2%)	748 (65.4%)	0.283
Median follow-up, months (IQR)	48 (22–76)	49 (23–77)	43 (20–69)	46 (22–71)	
Deaths	781 6.0%)	638 (6.0%)	72 (5.6%)	45 (3.9%)	
Breast cancer	409 (3.1%)	332 (3.1%)	52 (4.1%)	25 (2.2%)	
Other cause	372 (2.8%)	306 (2.9%)	20 (1.6%)	20 (1.7%)	

*AAPIs* Asian American/Pacific Islanders, *BCS* breast-conserving surgery, *ER* estrogen receptor, *HER2* human epidermal growth factor receptor 2, *HR* hormone receptor, *IDC* invasive ductal carcinoma, *ILC* invasive lobular carcinoma, *PR* progesterone receptor, *RS* recurrence score

### Comparing BCSM by treatment of chemotherapy in patients who had tumors with RS > 25

Among the 13,123 women who had an RS > 25, only 8,328 (63.5%) had received chemotherapy. Treatment with chemotherapy differed by age, tumor size, and histologic grade among this sub-cohort, although they all were candidates for adjuvant chemotherapy based on their 21-gene RS results (Table 1). Baseline characteristics of patients with or without chemotherapy are summarized in supplementary Table S2.

**Fig. 2:**
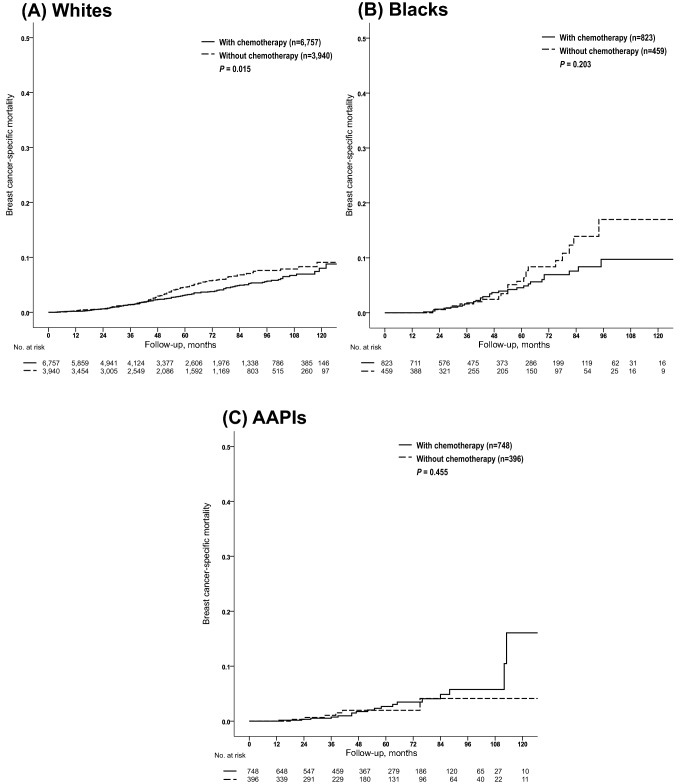
Breast cancer-specific mortality by treatment of chemotherapy within each racial group among the 21-gene RS-based high-risk group (RS > 25). *AAPIs* Asian Americans/Pacific islanders, *RS* recurrence score

For the entire sub-cohort, BCSM was significantly lower in patients who had received chemotherapy than in those who did not (unadjusted HR = 0.783; 95% CI, 0.644–0.952; *P* = 0.014). White women, who comprised the majority of this sub-cohort, showed a similar outcome compared to the entire sub-cohort (unadjusted HR = 0.766; 95% CI, 0.617–0.951; *P* = 0.016). Differences in BCSM between the two patient populations divided by receipt of chemotherapy were not significant among Blacks (unadjusted HR 0.701; 95% CI, 0.404–1.216; *P* = 0.206) and AAPIs (unadjusted HR = 1.395; 95% CI, 0.580–3.357; *P* = 0.457) (Fig. 2). In a multivariate model including age (< 50 vs. ≥ 50 years), T category (T1 vs. T2), histologic grade (G1 vs. G2 vs. G3), histologic type (IDC vs. ILC vs. IDC + ILC vs. others), HER2 status (negative vs. positive vs. unknown), and RT treatment (no vs. yes), BCSMs were significantly lower with chemotherapy than without the therapy for all women who had tumors with RS > 25 (adjusted HR = 0.756; 95% CI, 0.618–0.924; *P* = 0.006), and for the White sub-population (adjusted HR = 0.734; 95% CI, 0.588–0.917; *P* = 0.006). However, we did not observe any significant differences in BCSM between the two groups, categorized by whether they were treated with chemotherapy or not, among Blacks (adjusted HR = 0.748; 95% CI, 0.428–1.307; *P* = 0.308) and AAPIs (adjusted HR = 1.343; 95% CI, 0.558–3.233; *P* = 0.511) (Supplementary Table S3).

**Fig. 3:**
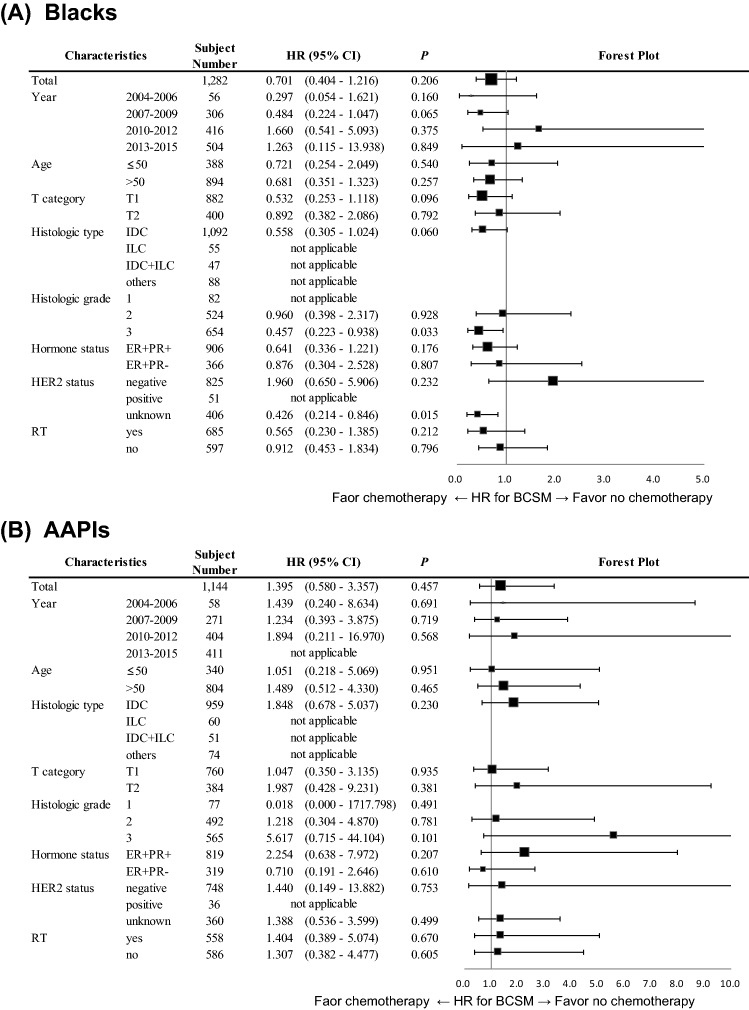
Forest plot demonstrating a comparison of BCSM by treatment of chemotherapy for different subgroups of **A** Blacks, **B** AAPIs. *AAPIs* Asian American/Pacific Islander, *BCSM* breast cancer-specific mortality, *CI* confidence interval, *ER* estrogen receptor, *HER2* human epidermal growth factor receptor 2, *HR* hazard ratio, *IDC* invasive ductal carcinoma, *ILC* invasive lobular carcinoma, *PR* progesterone receptor, *RT* radiotherapy

The outcomes were reevaluated within the subgroups subdivided by year of diagnosis, age, T category, histologic type and grade, HR status, HER2 status, and RT treatment. For White women, patients who received chemotherapy showed lower BCSM rates than those who did not in most subgroups (Supplementary Figure S2). However, these outcome differences were not evident within most subgroups of non-White women, except for Black women with grade 3 tumors (Fig. 3).

Relative hazard ratios for BCSM by chemotherapy treatment across RS risk categories within each racial group.

**Fig. 4:**
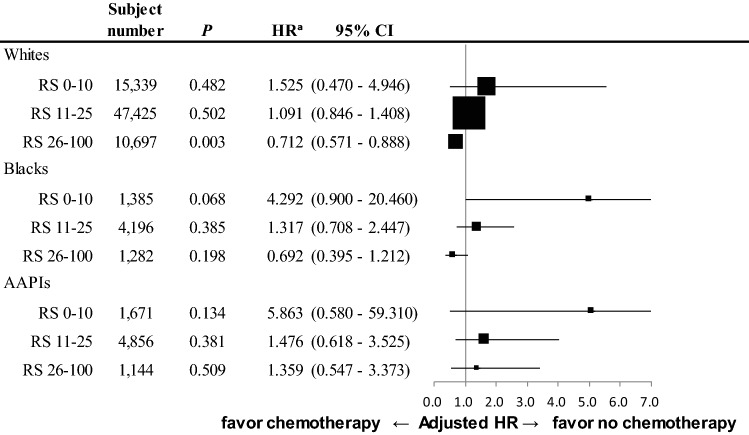
Risk of breast cancer-specific mortality by treatment of chemotherapy in each RS-based risk category/Race. *AAPIs* Asian American/Pacific Islander, *CI* confidence interval, *HER2* human epidermal growth factor receptor 2, *HR* hazard ratio, *RS* recurrence score. ^a^After adjustment of T category, histologic grade, HER2 status, and treatment of radiotherapy

Relative hazard ratios for BCSM were evaluated after adjusting for T category (T1 vs. T2), histologic grade (G1 vs. G2 vs. G3), HER2 status (negative vs. positive vs. unknown), and receipt of adjuvant radiotherapy (no vs. yes). Those adjusting variables were selected based on their consistently significant influence on the BCSM within the study cohort. Adjusted HR for BCSM by treatment with chemotherapy showed a serial trend of negative correlation with RS within Whites and Blacks. However, this trend was not definite in AAPIs across RS risk categories. We did not find any statistically significant differences in BCSM regardless of the RS risk categories for AAPIs (Fig. 4).

### Comparing BCSM among high-risk AAPIs after PS matching

To further evaluate the difference in BCSM among high-risk AAPIs (RS > 25), we performed PS matching to reduce bias related to the influence of patient and tumor characteristics on the decision to omit chemotherapy. PS matching was performed for 748 patients with chemotherapy and 396 patients without chemotherapy. The PSs were calculated using a logistic regression model with the following independent variables: age (≤ 50 vs. > 50 years), T category, histologic grade, hormonal status, HER2 status, type of breast surgery and receipt of radiotherapy. In total, we identified 748 PS-matched patients (374 patients with chemotherapy, 374 patients without chemotherapy). The characteristics before and after the PS matching are summarized in supplementary Table S4.

Among the 748 PS-matched patients, the adjusted HR for BCSM in patients with chemotherapy was 1.008 (95% CI 0.334–3.041). This result agrees with the result from the multivariable analysis. Supplementary Figure S3 shows unadjusted and adjusted HRs for BCSM in patients with chemotherapy among AAPIs who had a RS > 25.

### Sensitivity analysis with original categorization of high-risk RS group (RS > 30)

Based on the original criteria of the 21-gene RS assay, 6662 women were categorized into the high-risk RS group (RS > 30), which included 5376 Whites, 718 Blacks, and 568 AAPIs. For this sub-cohort, White women showed a consistently lower risk of BCSM with chemotherapy than without chemotherapy in the univariate and multivariate analyses. Black women showed a significantly lower risk of BCSM with chemotherapy despite the small number of patients in the univariate analysis (unadjusted HR = 0.503; 95% CI, 0.261–0.971; *P* = 0.041), although the difference in BCSM was not significant in the multivariate model. For AAPIs, we did not observe any significant difference in BCSM between the two groups, categorized by whether or not they were treated with chemotherapy (Supplementary Figure S4 and S5).

## Discussion

Our results revealed that within the 21-gene RS-based high-risk group, differences in BCSM between patients who had chemotherapy and those who did not were not as evident in Blacks and AAPIs as in Whites; this demonstrates that prediction of the benefits of chemotherapy based on the current 21-gene RS-based risk categorization among non-White women plays a poor role or, at most, an incomplete one. This is a considerable weakness given that the assay has already become popular in many Asian countries and, furthermore, might mislead to overtreatment with adjuvant chemotherapy.

NSABP B-20 and B-14 trials enrolled participants in the 1980s and early 1990s from institutions in the United States and Canada. The 21-gene RS assay development was based on the background information of tumors from a subset of participants from those trials, which was assumed to include only 5%–6% of data from black women. The contribution of data from Asians and other ethnic minority groups might likewise be small, given their small proportion in the background society. Differences in clinical and tumor characteristics between Asian and Western breast cancer patients have been documented in previous studies [5, 14]. Furthermore, a recent study revealed that there were significant outcome differences between races within the risk groups stratified by the 21-gene RS [15, 16]. These findings suggest that detailed genetic backgrounds of breast cancer might differ between races, although they have much in common across races.

On the other hand, evidence to validate the 21-gene RS assay in Asian populations is limited. Toi et al. reported a significant prognostic value of the assay in 200 Japanese patients with breast cancer [17]. However, another important role of the assay, which is predicting benefits from adjuvant chemotherapy, has not been validated in Asian populations.

Currently, several genetic assays for breast cancer are being tried to substitute the 21-gene RS assay, which is mainly based on Asian breast cancer cohort data. The Breast Cancer Test (BCT) score is a quantitative, real-time reverse transcription polymerase chain reaction-based multigene assay including 6 prognostic and 3 reference genes, which predicts the risk of distant recurrences and benefits from adjuvant chemotherapy among HR-positive/HER2-negative (HR + /HER2 −) EBC patients based on data from 906 and 346 Korean breast cancer cohorts, respectively [18–20]. OncoFREE is another next-generation sequencing (NGS)-based multigene assay that includes 21 prognostic and 158 reference genes, which presented a powerful prognostic value, especially among patients aged ≤ 50 years, in predicting the risk of distant recurrences from data of 343 HR + /HER2 − Korean EBC patients [21]. In Japan, a 95-gene signature was developed from Japanese HR + /HER2 − EBC cohort data, which suggested comparable and auxiliary utility to the 21-gene RS assay [22, 23]. Additionally, an 18-gene classifier, which developed from Chinese HR + /HER2 − EBC cohort data, has reported favorable preliminary results [24].

These assays might have an advantage in predicting the prognosis of Asian HR + /HER2 − EBC patients over the 21-gene RS assay, as their development was based on Asian data. It should be noted that each assay showed a comparable prognostic value with the 21-gene RS assay in predicting distant recurrences [20, 21], and one of them presented a significant capacity for categorizing HR + /HER2 − EBC patients classified by whether they experienced benefit from adjuvant chemotherapy or not [19], which was different from the incomplete role of the 21-gene RS assay in predicting chemotherapeutic benefits among AAPIs in this study. However, these new assays have limitations, when compared to the 21-gene RS assay, due to the small size of validation cohorts. Additionally, their validation cohorts were less balanced than those of the 21-gene RS assay because they were not selected from randomized controlled trials, but from institutional databases. Although the strategy of these new assays might not be the best, it could be a practical one, given that randomized controlled trials with long-term follow-up data are limited among Asian populations.

The current study had several limitations other than its intrinsic pitfalls because of its retrospective study design. The study endpoint was not a distant recurrence, but a breast cancer-related death, although the latter might reflect the former as a final result. The median follow-up was relatively short, given the intervals between recurrences in distal organs and deaths, which highlights the importance of prolonged follow-up with analysis of delayed events [25, 26]. Another limitation is the incomplete coding strategy for adjuvant therapy of the SEER registry, which did not discriminate patients whose receipt of adjuvant chemotherapy was uncertain from those who were not administered the therapy. This coding strategy may disguise the benefits of adjuvant chemotherapy during our analyses. Nevertheless, the current study had strength in evaluating the predictive value of the 21-gene RS assay for chemotherapeutic benefit based on the largest non-White cohort currently available. Given that little evidence is available for the current issue, this study provides an informative reference when making a decision to use the 21-gene RS as a predictor for chemotherapeutic benefit among non-White women. Furthermore, the smaller number of events in AAPIs (2.2% vs. 3.1% in Whites and 4.1% in Blacks) is an interesting point in itself because they were selected by the 21-gene RS stratification in the same way with White or Black women. The incomplete prediction of chemotherapeutic benefit among non-White women in our results might be a counterevidence supporting concerns about the relevance of the assay among those populations [27–30], because even the largest cohort data of non-White women from the SEER registry could not reveal that predictive value. Of note, further studies based on the larger number of subjects with direct recurrence data should be warranted to confirm the predictive value of the 21-gene RS assay in AAPIs, because our result might be limited by a small number of events and the indirect study endpoint, BCSM instead of recurrence.

In conclusion, the 21-gene RS assay did not reveal its known predictive value of categorizing patients among the non-White HR-positive EBC population based on the analyses of the SEER registry, according to whether they benefited from adjuvant chemotherapy or not. Given that the assay is widely used for Asian EBC patients, proper validation of its predictive value in the selection of adjuvant chemotherapy is warranted.

## Supplementary Information

Below is the link to the electronic supplementary material.

Supplementary file1 (DOCX 2234 KB)

## Data Availability

The datasets supporting the findings of this study were extracted from the population-based SEER Oncotype DX Database (https://seer.cancer.gov/seerstat/databases/oncotype-dx/index.html). A metadata record describing the datasets generated and analyzed during the current study is available from the corresponding author on reasonable request
