# Supplementary material for: Racial differences in predictive value of the 21-gene recurrence score assay: a population-based study using the SEER database
Source: Breast Cancer. 2022 May 26;29(5):889–98. doi: 10.1007/s12282-022-01371-z (PMC9385768; doi:10.1007/s12282-022-01371-z)
Supplement: Supplementary file 1 — Supplementary file1 (DOCX 2234 KB) [file 12282_2022_1371_MOESM1_ESM.docx]

**Table S1** Composition of Asian Americans/Pacific islanders in the SEER database

| **Race/Ethnicity** | **number** | **%** |
| --- | --- | --- |
| Filipino | 284 | 24.8 |
| Chinese | 178 | 15.6 |
| Japanese | 131 | 11.5 |
| Asian Indian or Pakistani | 97 | 8.5 |
| Vietnamese | 67 | 5.9 |
| Korean | 65 | 5.7 |
| American Indian/Alaska Native | 65 | 5.7 |
| Hawaiian | 52 | 4.5 |
| Thai | 14 | 1.2 |
| Pacific Islander | 12 | 1.0 |
| Samoan | 8 | 0.7 |
| Kampuchean | 4 | 0.3 |
| Tongan | 2 | 0.2 |
| Fiji Islander | 2 | 0.2 |
| Guamanian | 2 | 0.2 |
| Chamorran | 1 | 0.1 |
| Laotian | 1 | 0.1 |
| Melanesian | 1 | 0.1 |
| Micronesian | 1 | 0.1 |
| Polynesian | 1 | 0.1 |
| Tahitian | 1 | 0.1 |
| Other Asian | 155 | 13.5 |
| **Total** | **1144** | **100.0** |

**Table S2** Baseline characteristics of patients with / without chemotherapy in each racial group among the 21−gene RS−based high-risk group (RS>25) selected from the SEER database

|  |  |  | **White (n = 10,697)** | | | | **Black (n = 1,282)** | | | | **AAPI (n = 1,144)** | | | |
| --- | --- | --- | --- | --- | --- | --- | --- | --- | --- | --- | --- | --- | --- | --- |
|  |  |  | Chemotherapy | | No Chemotherapy | | Chemotherapy | | No Chemotherapy | | Chemotherapy | | No Chemotherapy | |
|  | | | 6,757 | (63.2%) | 3,940 | (36.8%) | 823 | (64.2%) | 459 | (35.8%) | 748 | (65.4%) | 396 | (34.6%) |
| Year of diagnosis | |  |  |  |  |  |  |  |  |  |  |  |  |  |
|  | 2004-2006 | | 473 | (7.0%) | 329 | (8.4%) | 35 | (4.3%) | 21 | (4.6%) | 30 | (4.0%) | 28 | (7.1%) |
|  | 2007-2009 | | 1,743 | (25.8%) | 1,040 | (26.4%) | 194 | (23.6%) | 112 | (24.4%) | 184 | (24.6%) | 87 | (22.0%) |
|  | 2010-2012 | | 2,119 | (31.4%) | 1,336 | (33.9%) | 273 | (33.2%) | 143 | (31.2%) | 268 | (35.8%) | 136 | (34.3%) |
|  | 2013-2015 | | 2,422 | (35.8%) | 1,235 | (31.3%) | 321 | (39.0%) | 183 | (39.9%) | 266 | (35.6%) | 145 | (36.6%) |
| Patient age, years (range) | | | 58 (19-87) | | 62 (18-91) | | 55 (24-88) | | 60 (26-92) | | 55 (25-81) | | 60 (21-87) | |
|  | ≤50 | | 1,768 | (26.2%) | 669 | (17.0%) | 292 | (35.5%) | 96 | (20.9%) | 263 | (35.2%) | 77 | (19.4%) |
|  | >50 | | 4,989 | (73.8%) | 3,271 | (83.0%) | 531 | (64.5%) | 363 | (79.1%) | 485 | (64.8%) | 319 | (80.6%) |
| T category | | |  |  |  |  |  |  |  |  |  |  |  |  |
|  | T1 | | 4,742 | (70.2%) | 2,886 | (73.2%) | 556 | (67.6%) | 326 | (71.0%) | 487 | (65.1%) | 273 | (68.9%) |
|  | T2 | | 2,015 | (29.8%) | 1,054 | (26.8%) | 267 | (32.4%) | 133 | (29.0%) | 261 | (34.9%) | 123 | (31.1%) |
| Surgery type | | |  |  |  |  |  |  |  |  |  |  |  |  |
|  | BCS | | 4,585 | (67.9%) | 2,566 | (65.1%) | 580 | (70.5%) | 296 | (64.5%) | 465 | (62.2%) | 213 | (53.8%) |
|  | Mastectomy | | 2,172 | (32.1%) | 1,374 | (34.9%) | 243 | (29.5%) | 163 | (35.5%) | 283 | (37.8%) | 183 | (46.2%) |
| Histologic type | | |  |  |  |  |  |  |  |  |  |  |  |  |
|  | IDC | | 5,675 | (84.0%) | 3,172 | (80.5%) | 705 | (85.7%) | 387 | (84.3%) | 627 | (83.8%) | 332 | (83.8%) |
|  | ILC | | 359 | (5.3%) | 295 | (7.5%) | 29 | (3.5%) | 26 | (5.7%) | 39 | (5.2%) | 21 | (5.3%) |
|  | IDC and ILC | | 323 | (4.8%) | 254 | (6.4%) | 31 | (3.8%) | 16 | (3.5%) | 33 | (4.4%) | 18 | (4.5%) |
|  | others | | 400 | (5.9%) | 219 | (5.6%) | 58 | (7.0%) | 30 | (6.5%) | 49 | (6.6%) | 25 | (6.3%) |
| Histologic grade | | |  |  |  |  |  |  |  |  |  |  |  |  |
|  | 1 | | 465 | (6.9%) | 494 | (12.5%) | 36 | (4.4%) | 46 | (10.0%) | 37 | (4.9%) | 40 | (10.1%) |
|  | 2 | | 2,839 | (42.0%) | 1,870 | (47.5%) | 321 | (39.0%) | 203 | (44.2%) | 302 | (40.4%) | 190 | (48.0%) |
|  | 3 | | 3,350 | (49.6%) | 1,498 | (38.0%) | 455 | (55.3%) | 199 | (43.4%) | 404 | (54.0%) | 161 | (40.7%) |
|  | unknown | | 103 | (1.5%) | 78 | (2.0%) | 11 | (1.3%) | 11 | (2.4%) | 5 | (0.7%) | 5 | (1.3%) |
| HR status | | |  |  |  |  |  |  |  |  |  |  |  |  |
|  | ER+ PR+ | | 4,900 | (72.5%) | 2,862 | (72.6%) | 594 | (72.2%) | 312 | (68.0%) | 546 | (73.0%) | 273 | (68.9%) |
|  | ER+ PR- | | 1,819 | (26.9%) | 1,046 | (26.5%) | 224 | (27.2%) | 142 | (30.9%) | 199 | (26.6%) | 120 | (30.3%) |
|  | ER+ unknown PR | | 14 | (0.2%) | 6 | (0.2%) | 5 | (0.6%) | 5 | (1.1%) |  |  | 2 | (0.5%) |
|  | ER- PR+ | | 24 | (0.4%) | 26 | (0.7%) |  |  |  |  | 3 | (0.4%) | 1 | (0.3%) |
| HER2 status | | |  |  |  |  |  |  |  |  |  |  |  |  |
|  | positive | | 231 | (3.4%) | 115 | (2.9%) | 36 | (4.4%) | 15 | (3.3%) | 26 | (3.5%) | 10 | (2.5%) |
|  | negative | | 4,164 | (61.6%) | 2,344 | (59.5%) | 531 | (64.5%) | 294 | (64.1%) | 492 | (65.8%) | 256 | (64.6%) |
|  | unknown | | 2,362 | (35.0%) | 1,481 | (37.6%) | 256 | (31.1%) | 150 | (32.7%) | 230 | (30.7%) | 130 | (32.8%) |
| Radiotherapy | | | 3,899 | (57.7%) | 1,819 | (46.2%) | 483 | (58.7%) | 202 | (44.0%) | 407 | (54.4%) | 151 | (38.1%) |
| Median follow-up, months (IQR) | | | 47 (22-77) | | 50 (25-77) | | 43 (19-70) | | 42 (20-67) | | 46 (22-71) | | 44 (22-71) | |
| Deaths | | | 327 | (4.8%) | 311 | (4.6%) | 51 | (6.2%) | 47 | (10.2%) | 31 | (4.1%) | 14 | (3.5%) |
|  | breast cancer | | 185 | (2.7%) | 147 | (2.2%) | 30 | (3.6%) | 22 | (4.8%) | 18 | (2.4%) | 7 | (1.8%) |
|  | other cause | | 142 | (2.1%) | 164 | (2.4%) | 21 | (2.6%) | 25 | (5.4%) | 13 | (1.7%) | 7 | (1.8%) |

*AAPIs* Asian American/Pacific Islanders, *BCS* breast-conserving surgery, *ER* estrogen receptor, *HER2* human epidermal growth factor receptor 2, *HR* hormone receptor, *IDC* invasive ductal carcinoma, *ILC* invasive lobular carcinoma, *PR* progesterone receptor, *RS* recurrence score

**Table S3** Multivariate analysis for breast cancer-specific mortality in each racial group among the 21−gene RS−based high-risk group (RS>25) selected from the SEER database

|  | **Whites (n=10,697)** | | | | | | | | **Blacks (n=1,282)** | | | | | | | | | | **AAPIs (n=1,144)** | | | | | | | | | | |
| --- | --- | --- | --- | --- | --- | --- | --- | --- | --- | --- | --- | --- | --- | --- | --- | --- | --- | --- | --- | --- | --- | --- | --- | --- | --- | --- | --- | --- | --- |
| **Variable** | **Univariate analysis** | | | | **Multivariate analysisᵃ** | | | | **Univariate analysis** | | | | **Multivariate analysisᵃ** | | | | | | **Univariate analysis** | | | | | | **Multivariate analysisᵃ** | | | | |
|  | **HR (95% CI)** | | | ***P*** | **HR (95% CI)** | | | ***P*** | **HR (95% CI)** | | | ***P*** | **HR (95% CI)** | | | | ***P*** | | **HR (95% CI)** | | | | ***P*** | | **HR (95% CI)** | | | | ***P*** |
| Adjuvant chemotherapy |  |  |  |  |  |  |  |  |  |  |  |  |  |  |  |  | |  | |  |  |  | |  | |  |  |  | |
| yes vs. no | 0.766 | (0.617 − 0.951) | | 0.016 | 0.734 | (0.588 − 0.917) | | 0.006 | 0.701 | (0.404 − 1.216) | | 0.206 | 0.748 | (0.428 − 1.307) | | 0.308 | | 1.395 | | (0.580 − 3.357) | | 0.457 | | 1.343 | | (0.558 − 3.233) | | 0.511 | |
| Age |  |  |  |  |  |  |  |  |  |  |  |  |  |  |  |  | |  | |  |  |  | |  | |  |  |  | |
| >50 vs. ≤50 | 1.398 | (1.066 − 1.833) | | 0.016 | 1.289 | (0.979 − 1.698) | | 0.071 | NS | | | |  |  |  |  | | NS | | | | | |  | |  |  |  | |
| T category |  |  |  |  |  |  |  |  |  |  |  |  |  |  |  |  | |  | |  |  |  | |  | |  |  |  | |
| T2 vs. T1 | 2.408 | (1.939 − 2.989) | | <0.001 | 2.212 | (1.774 − 2.757) | | <0.001 | 2.135 | (1.237 − 3.684) | | 0.006 | 2.016 | (1.163 − 3.496) | | 0.013 | | 1.968 | | (0.885 − 4.375) | | 0.097 | | 2.197 | | (0.985 − 4.900) | | 0.054 | |
| Histologic grade |  |  |  |  |  |  |  |  |  |  |  |  |  |  |  |  | |  | |  |  |  | |  | |  |  |  | |
| 2 vs. 1 | 2.079 | (1.148 − 3.764) | | 0.016 | 1.913 | (1.053 − 3.474) | | 0.033 | NS | | | |  |  |  |  | | NS | | | | | |  | |  |  |  | |
| 3 vs. 1 | 3.444 | (1.922 – 6.171) | | <0.001 | 2.962 | (1.637 − 5.360) | | <0.001 | NS | | | |  |  |  |  | | NS | | | | | |  | |  |  |  | |
| Histologic type |  |  |  |  |  |  |  |  |  |  |  |  |  |  |  |  | |  | |  |  |  | |  | |  |  |  | |
| ILC vs. IDC | 1.032 | (0.663 − 1.608) | | 0.889 | 1.064 | (0.668 − 1.695) | | 0.795 | 1.282 | (0.310 − 5.306) | | 0.731 | 1.133 | (0.273 − 4.707) | | 0.864 | | NS | | | | | |  | |  |  |  | |
| IDC+ILC vs. IDC | 0.779 | (0.463 − 1.309) | | 0.345 | ` | (0.468 − 1.329) | | 0.373 | 2.418 | (0.956 − 6.114) | | 0.062 | 2.080 | (0.819 − 5.282) | | 0.124 | | NS | | | | | |  | |  |  |  | |
| others vs. IDC | 0.549 | (0.301 − 1.003) | | 0.051 | 0.584 | (0.319 − 1.070) | | 0.082 | 0.802 | (0.249 − 2.590) | | 0.713 | 0.809 | (0.250 − 2.617) | | 0.724 | | NS | | | | | |  | |  |  |  | |
| HER2 status |  |  |  |  |  |  |  |  |  |  |  |  |  |  |  |  | |  | |  |  |  | |  | |  |  |  | |
| positive vs. negative | 1.282 | (0.993 − 1.654) | | 0.057 | 1.294 | (0.656 − 2.552) | | 0.457 | NS | | | |  |  |  |  | | 0.332 | | (0.107 − 1.033) | | 0.057 | | NS | | | | 0.977 | |
| unknown vs. negative | 1.611 | (0.819 − 3.170) | | 0.167 | 0.818 | (0.633 − 1.057) | | 0.124 | NS | | | |  |  |  |  | |  | |  |  |  | | 3.415 | | (1.092 − 10.682) | | 0.035 | |
| Adjuvant radiotherapy |  |  |  |  |  |  |  |  |  |  |  |  |  |  |  |  | |  | |  |  |  | |  | |  |  |  | |
| yes vs. no | 0.807 | (0.651 − 1.001) | | 0.051 | 0.909 | (0.730 − 1.133) | | 0.396 | 0.531 | (0.304 − 0.929) | | 0.026 | 0.592 | (0.335 − 1.045) | | 0.071 | | NS | | | | | |  | |  |  |  | |

*AAPIs* Asian American/Pacific Islander, *CI* confidence interval, *HER2* human epidermal growth factor receptor 2, *HR* hazard ratio, *IDC* invasive ductal carcinoma, *ILC* invasive lobular carcinoma, *NS* not significant, *PR* progesterone receptor, *RS* recurrence score

ᵃVariables which have *P*-value <0.1 in univariate analyses were inputted into multivariate analysis

**Table S4.** Characteristics of patients with or without chemotherapy before and after propensity score matching for AAPIs who had a RS>25

|  |  | **Before matching** | | **After matching** | |
| --- | --- | --- | --- | --- | --- |
|  |  | **Chemotherapy** | **No Chemotherapy** | **Chemotherapy** | **No Chemotherapy** |
|  |  | **(N=748)** | **(N=396)** | **(N=374)** | **(N=374)** |
| Age | |  |  |  |  |
|  | ≤50 | 263 (35.2%) | 77 (19.4%) | 74 (19.8%) | 76 (20.3%) |
|  | >50 | 485 (64.8%) | 319 (80.6%) | 300 (80.2%) | 298 (79.7%) |
| T category | |  |  |  |  |
|  | T1 | 487 (65.1%) | 273 (68.9%) | 260 (69.5%) | 259 (69.3%) |
|  | T2 | 261 (34.9%) | 123 (31.1%) | 114 (30.5%) | 115 (30.7%) |
| Histologic grade | |  |  |  |  |
|  | 1 | 37 (4.9%) | 40 (10.1%) | 30 (8.0%) | 34 (9.1%) |
|  | 2 | 302 (40.4%) | 190 (48.0%) | 184 (49.2%) | 178 (47.6%) |
|  | 3 | 404 (54.0%) | 161 (40.7%) | 158 (42.2%) | 159 (42.5%) |
|  | Unknown | 5 (0.7%) | 5 (1.3%) | 2 (0.5%) | 3 (0.8%) |
| HR status | |  |  |  |  |
|  | ER+, PR+ | 546 (73.0%) | 273 (68.9%) | 261 (69.8%) | 260 (69.5%) |
|  | ER+, PR- | 199 (26.6%) | 120 (30.3%) | 113 (30.2%) | 113 (30.2%) |
|  | ER+, PR unknown |  | 2 (0.5%) |  |  |
|  | ER-, PR+ | 3 (0.4%) | 1 (0.3%) |  | 1 (0.3%) |
| Surgery type | |  |  |  |  |
|  | BCS | 465 (62.2%) | 213 (53.8%) | 206 (55.1%) | 205 (54.8%) |
|  | Mastectomy | 283 (37.8%) | 183 (46.2%) | 168 (44.9%) | 169 (45.2%) |
| HER2 status | |  |  |  |  |
|  | Positive | 492 (65.8%) | 256 (64.6%) | 9 (2.4%) | 10 (2.7%) |
|  | Negative | 26 (3.5%) | 10 (2.5%) | 249 (66.6%) | 248 (66.3%) |
|  | Unknown | 230 (30.75) | 130 (32.8%) | 116 (31.0%) | 116 (31.0%) |
| Radiotherapy | |  |  |  |  |
|  | No | 341 (45.6%) | 245 (61.9%) | 222 (59.4%) | 224 (59.9%) |
|  | Yes | 407 (54.4%) | 151 (38.1%) | 152 (40.6%) | 150 (40.1%) |

*AAPI* Asian American/Pacific Islander, *BCS* breast conserving surgery, *ER* estrogen receptor, *HER2* human epidermal growth factor receptor 2, *PR* progesterone receptor, *RS* recurrence score

**Fig. S1** Selection of study population from the SEER Oncotype DX database

*ER* estrogen receptor, *PR* progesterone receptor, *RS* recurrence score

**Fig. S2** Forest plot demonstrating a comparison of BCSM by treatment of chemotherapy for different subgroups of Whites

*BCSM* breast cancer-specific mortality, *CI* confidence interval, *ER* estrogen receptor, *HER2* human epidermal growth factor receptor 2, *HR* hazard ratio, *IDC* invasive ductal carcinoma, *ILC* invasive lobular carcinoma, *PR* progesterone receptor, *RT* radiotherapy

**Fig. S3** Unadjusted and adjusted risk of breast cancer-specific mortality by treatment of chemotherapy in AAPIs who had RS>25

*AAPIs* Asian American/Pacific Islander, *CI* confidence interval, *HER2* human epidermal growth factor receptor 2, *HR* hazard ratio, *PS* propensity score, *RS* recurrence score.

ᵃAfter adjustment of T category, HER2 status

ᵇAfter matching of PS calculated by a model including the following variables: age (≤50 vs. >50 years), T category, histologic grade, hormonal status, HER2 status, type of breast surgery and receipt of radiotherapy

**Fig. S4** Breast cancer-specific mortality by treatment of chemotherapy within each racial group among the patients with tumors with RS>30.

*AAPIs* Asian Americans/Pacific islanders, *RS* recurrence score

**Fig. S5** Risk of breast cancer-specific mortality by treatment of chemotherapy in each RS−based risk category (by original categorization)/Race

*AAPIs* Asian American/Pacific Islander, *CI* confidence interval, *HER2* human epidermal growth factor receptor 2, *HR* hazard ratio, *RS* recurrence score.

ᵃAfter adjustment of T category, histologic grade, HER2 status, and treatment of radiotherapy
